# Pain Education in the Management of Patients with Chronic Low Back Pain: A Systematic Review

**DOI:** 10.3390/jfmk7040074

**Published:** 2022-09-26

**Authors:** Rosario Ferlito, Chiara Blatti, Ludovico Lucenti, Umberto Boscarino, Marco Sapienza, Vito Pavone, Gianluca Testa

**Affiliations:** 1 Department of Biomedical and Biotechnological Sciences, University of Catania, 95123 Catania, Italy; 2 Department of General Surgery and Medical-Surgical Specialties, A.O.U. Policlinico Rodolico-San Marco, University of Catania, Via Santa Sofia, 78, 95123 Catania, Italy

**Keywords:** pain education, cognitive behavioral therapy, chronic low back pain, chronic lumbar pain

## Abstract

New prospective of chronic low back pain (CLBP) management based on the biopsychosocial model suggests the use of pain education, or neurophysiological pain education, to modify erroneous conceptions of disease and pain, often influenced by fear, anxiety and negative attitudes. The aim of the study is to highlight the evidence on the outcomes of a pain education-oriented approach for the management of CLBP. The search was conducted on the Pubmed, Scopus, Pedro and Cochrane Library databases, leading to 2673 results until September 2021. In total, 13 articles published in the last 10 years were selected as eligible. A total of 6 out of 13 studies support a significant reduction in symptoms in the medium term. Disability is investigated in only 11 of the selected studies, but 7 studies support a clear reduction in the medium-term disability index. It is difficult to assess the effectiveness of the treatments of pain education in patients affected by CLBP, due to the multimodality and heterogeneity of the treatments administered to the experimental group. In general, methods based on pain education or on cognitive-behavioral approaches, in association with physical therapy, appear to be superior to physiotherapeutic interventions alone in the medium term.

## 1. Introduction

### 1.1. Pathology and Intervention

The role of psychological factors in the development and persistence of chronic low back pain (CLBP) [1] was largely investigated in the recent literature. In particular, studies have suggested that an increasing negative attitude towards pain, fear of movement or relapses, plays an important role in the etiology of chronic low back pain [2].

Chronic low back pain is one of the most significant and frequent health problems, characterized by medical and economic consequences for the patients themselves and for society, such as increased medical expenses, lost income, lost productivity and a reduction on compensation payments.

The approach to chronic low back pain is multidisciplinary in terms of diagnostic and therapeutic viewpoints.

Medical, paramedical, physiotherapeutic, psychological and holistic methods are all useful in the best approach to this complex illness and to fully understand and treat all of the dimensions and aspects of discomfort felt by the patients.

According to the biopsychosocial model, chronic pain is mainly caused by a nervous system hypersensitivity, rather than the persistence of a lesion at the tissue level [3]. This neuronal hyperexcitability, which in turn causes a lower pain threshold, is due to a plasticity mechanism (known as central sensitization) [4], that is sustained by negative emotions, anxiety, fear, catastrophe and the anticipation of consequences [5]. Therefore, the most recent literature has suggested the use of pain education as a modality to treat chronic pain, particularly in clinical situations characterized by central sensitization, or in the presence of disease and/or pain misconceptions [6].

Lately, one of the most studied and utilized psychotherapeutic methods is cognitive behavioral therapy (CBT), which has been largely supported and aims to explore the links between thoughts, emotions and behaviors. It is a structured approach used to treat some mental health disorders and other illnesses with the aim to alleviate distress by helping patients to develop more adaptive cognitions and behaviors.

Pain education (Pain Neuroscience Education, PNE) is a treatment that consists of educational sessions, especially (but not only) for patients affected by musculoskeletal disorders, aimed at an accurate explanation of the neurophysiology and neurobiology of pain and the process of pain modulation by the central nervous system [7]. The goal is to modify those beliefs, rooted in the psychosocial background of the patient, which feed the persistence of chronic pain, remodeling the perception of pain itself and to draw positive effects, also in functional terms.

### 1.2. Objective

The purpose of this systematic review is to highlight the most recent scientific evidence on the outcomes of a pain-oriented approach in the management of Chronic Low Back Pain (CLBP).

This paper examines the clinical trials of the last years that carried out pain education/cognitive-behavioral therapy interventions on patients with CLBP and then compared with conventional physiotherapy approaches.

## 2. Methods

### 2.1. Protocol

PRISMA (Preferred Reporting Items for Systematic Reviews and Meta-Analyzes) guidelines were followed [8].

### 2.2. Inclusion and Exclusion Criteria

#### 2.2.1. Types of Studies

Types of studies included were clinical trials (CT) and randomized controlled trials (RCT), with the aim of evaluation of the efficacy of treatments focused on pain education for the management of CLBP. Only articles published in the last 10 years (from 2011 to 2021) were included. No a priori restrictions were set with respect to number of participants, participants’ assignation, randomization units, number of centers involved or consideration of participant preferences.

#### 2.2.2. Types of Participants

Studies with patients affected by CLBP were included. A temporal threshold of persistence of pain equal to or more than 3 months was established to consider low back pain as chronic, according to the literature by most of the authors [2].

##### Inclusion Criteria

Studies involving the use of pain education (neurophysiological education of pain) or communicative-educational interventions, such as cognitive behavioral therapy (CBT) or cognitive functional therapy (CFT), as a single intervention or combined with physiotherapeutic treatments.

- Studies that admitted, as elements of comparison, the conventional low back pain physiotherapeutic protocols.
- Studies that presented additional intervention groups (in addition to the one identified as the experimental group and the control group).

##### Exclusion Criteria

- Back pain, post-operative lumbar pain and lumbar pain related to specific pathologies;
- Populations with individuals under the age of 18;
- Cardiovascular, psychiatric, rheumatic, neoplastic or inflammatory pathologies.
- Studies that comprised additional pharmacological, instrumental or other interventions, not attributable to physiotherapy techniques.

#### 2.2.3. Types of Outcome

The main outcomes evaluated for eligibility were pain and/or disability.

### 2.3. Bibliographic Research

The databases “PubMed”, “Scopus”, “Pedro” and “Cochrane Library” were systematically reviewed by three independent authors (RF, UB and FP) from 30th September 2021, according to the guidelines of the Preferred Reporting Items for Systematic Reviews and Meta-Analyzes (PRISMA).

The keywords “pain education” “cognitive behavioral therapy”, “chronic low back pain” and “chronic lumbar pain” were used for the research. The aforementioned keywords were also searched in the form of mesh terms, and were combined using Boolean operators (“AND”, “OR” and “NOT”) in line with the clinical research question, according to the PICO model [9].The search strings produced are shown in the following table (Table 1). The search produced 2673 results, summed up across the various databases. Two independent reviewers (RF and UB) performed a study quality assessment. Any conflicts were resolved by consulting an additional author (FP)

## 3. Results

### 3.1. Selection of the Articles

**Figure 1:**
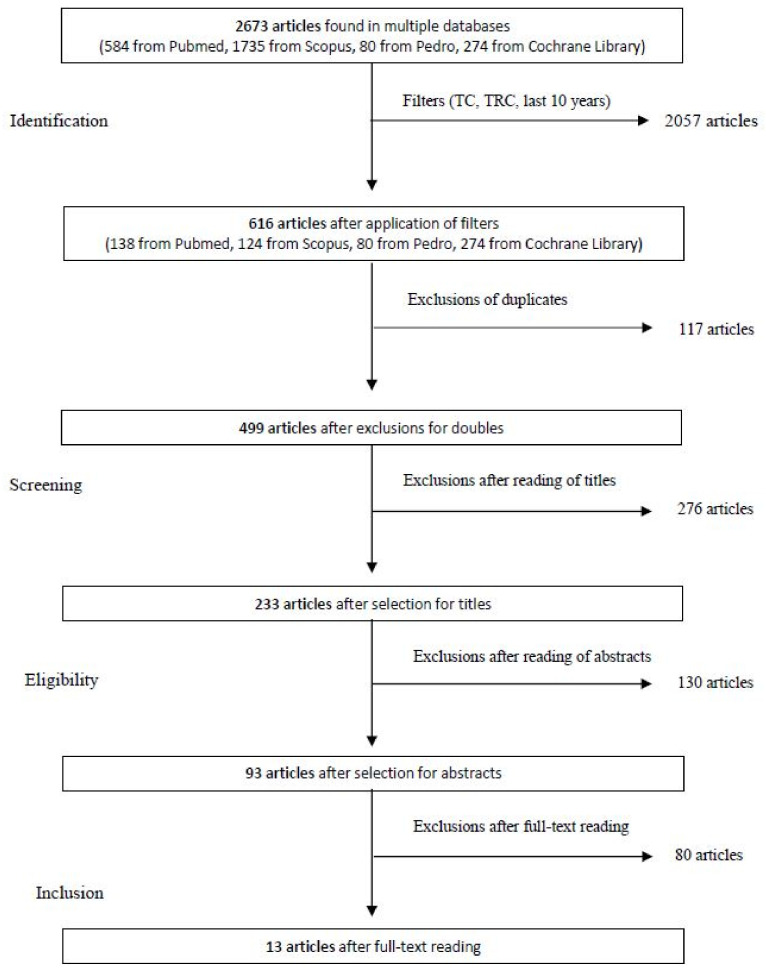
Flow chart of the study selection process.

Following the selection made through the filters (CT, CRT and publication in the last 10 years), the identified articles were reduced to 616, divided between Pubmed (138 articles), Scopus (124 articles), Pedro (80 articles) and Cochrane Library (274 articles), then further reduced to 499, after the exclusion of duplicates (117 articles). At this point, the qualifications were screened and 276 articles that did not show relevance to the research question were excluded. The remaining 223 articles were submitted for abstract reading, which excluded a further 130 articles, in favor of 93 eligible articles. A final selection was performed on these articles, reading the full text, and 80 more were excluded, due to the already cited exclusion criteria (Figure 1).

### 3.2. Bias Risk Assessment in the Included Studies

The assessment of the risk of bias in the studies included in this systematic review was carried out by two authors (RF and UB) using the PEDro scale; this tool allowed us to quickly identify which randomized clinical trials had internal validity (criteria 2–9) and had sufficient statistical information to make the results interpretable (criteria 10–11).

Any conflicts between the two authors were resolved through the comparison or intervention of a third author (FP).

Criterion 1, correlated with external validity (or “generability” or “applicability”), was, however, not used to calculate the total score [10].

The criteria that met each item of the PEDro scale are shown in Table 2:

The following tables show the results of the evaluation according to the PEDro scale for each study (Table 3). Afterwards, the results were summarized and expressed according to the corresponding levels of evidence (LOE) (Table 4).

Based on the results from the PEDro scale data, almost all studies were judged to be at low risk of bias for most of the items. Two studies were considered at high evidence level (level I) [21,23], nine studies at evidence level II [11,12,13,15,16,17,19,20,22] and two (one of which has a quasi-experimental design) with a low level of evidence [14,15].

### 3.3. Evaluation of External Validity or “Applicability”

Although the inclusion criteria identified studies with rather specific characteristics, with respect to the clinical presentation of symptoms, the duration of symptoms, the age group of the population and the type of interventions administered, there were still discrepancies between the studies. These discrepancies limit the possibility of drawing definitive conclusions about the efficacy of the treatment on the population under examination.

The settings where the studies were carried out were not considered (outpatient regime, hospital, university, specialized pain clinics or nursing homes).

Methods of administering the interventions (both experimental and control) were not homogenous in the programs offered, especially for multimodal interventions (a combination of educational approaches and physiotherapeutic treatments of different types), which made it impossible to determine the real knowledge of the effectiveness of individual treatments. Two studies [7,10] focused on the prevention of all the studies to overlap in a coherent way and evaluated pain as an outcome measure in the subsequent follow-up endpoints, but not the disability index, despite the latter being detected at baseline.

Finally, it is worth noting that the follow-up durations are rather heterogeneous between the studies, constituting a significant uncertainty in identifying the efficacy of the treatment with respect to short (<3 months), medium (from 3 months to 1 year) or long (≥1 year) term.

### 3.4. Extractions and Characteristics of Datas

In order to extract data from each article independently, a standard data extraction system was used in line with the PICO model of the clinical research question. The data, including the scores relating to the PEDro score and the LOEs of each study, previously obtained, were then organized according to the following parameters, and finally reported in the table (Table 5):

- General information: Author, year of publication, study design and level of evidence of the study;
- Participants: sample size, age of participants and duration of pain;
- Interventions/Controls: number of participants for each group (experimental and control), content, number of interventions;
- Outcome: type of outcome taken into consideration;
- Follow-up(s): baseline, post-treatment and re-evaluations;
- Results: summary of the results obtained, with mean difference (and standard deviation);
- Score on the PEDro scale.

## 4. Discussion

The aim of this systematic review was to provide the state of the art scientific literature regarding the efficacy of educational techniques in patients with CLBP, based on outcomes related to pain intensity and disability. The 13 included studies (12 CRTs) worked on 1641 participants. The results of the 13 studies were discussed separately, depending on the outcome measures investigated. According to pain reduction, investigated through VAS, NRS, NRS-11, PBI and CPAQ, six studies [11,12,14,18,19,23] out of thirteen significantly supported more evidence in the experimental group than in the control group. Moreover, in favor of the experimental group and to the detriment of the control group, another study [23] showed modest improvements, and one [17] showed a moderate reduction in painful symptoms detected after surgery, but which was not maintained at subsequent endpoints of follow-up. With regard to disability, measured by RDQ, mRDQ, ODI, HFAQ and QBPD, it must be highlighted that only 11 of the 13 included articles investigated this outcome measure, but seven studies supported an evident reduction in the disability index, in favor of the experimental group over the control group [11,12,13,16,18,19,22]. In the remaining studies, improvements were observed under the considered outcome measures, but without highlighting the significant differences between the experimental and control groups. The result would, therefore, be a success rate of the experimental intervention on the control group of 46.2% in relation to the reduction of pain, and of 63.7% in terms of improvements of disability. It is not possible to interpret these percentages in an absolute way, since, as has already been stated in the evaluation of external validity, the durations of the follow-ups were heterogeneous between the studies. For this reason, it was necessary to compare the data obtained from the previous estimates with the duration of the follow-ups that the various studies followed. Therefore, in relation to the endpoints of the studies, it emerged that the duration of the follow-ups was by far the medium term (considering the interval 3 months–1 year). This data, in particular, was found in all six studies (100%) that validated the success of the experimental intervention concerning the pain outcome, and in six [11,12,16,18,19,22] of the seven studies (85.7%) that supported the success of the experimental intervention on the reduction of disability. Another important note, also mentioned in the chapter on applicability, is the impossibility of rigorously drawing conclusions on the effectiveness of the intervention chosen by the clinical research question, due to the different formats used for the administration of the various types of intervention. The interventions performed in the experimental groups, in fact, encompass as a whole: pain education, pain adaptation strategies, CBT, relaxation techniques, cognitive restructuring, reorientation of objectives, deviation of attention, functional education, coping strategies, exposure with pain control and lifestyle change, physiotherapy, manual therapy techniques, Mulligan mobilizations, joint mobilizations, strengthening exercises, motor or sensory-motor control exercises, aerobic exercise, stretching and home physical activity combined in different formats. Similarly, for the control groups, the interventions embraced “packages” with the following variables: physiotherapy, manual therapy techniques, Mulligan mobilizations, joint mobilizations, strengthening exercises, motor control or sensory-motor exercises, aerobic exercise, stretching and physical activity at home. According to the data processed and weighted through the criteria mentioned above, the experimental interventions, usually combined with physiotherapeutic interventions of various types, show fair evidence of success in the medium term, relative to the two established outcomes, in comparison with conventional physiotherapeutic interventions, on patients with CLBP.

### Limits of the Study

The most frequent methodological limit concerns the impossibility of obtaining a blind of the subjects and operators, obviously due to the intervention modality chosen by the research question, which provides, in most cases, a face-to-face comparison between patient and therapist. Another element that constitutes a source of bias, found quite frequently, was the analysis by the intention to treatment, a criterion that is at high risk of bias in seven out of the thirteen studies examined. However, the studies in question were also taken into consideration because they are of considerable interest to the research question.

## 5. Conclusions

It appears difficult to express categorically the efficacy of treatment focused on pain education, or, more broadly, on cognitive behavioral therapy or cognitive functional therapy for patients with CLBP. However, it is possible to state that, based on what was filtered by the studies analyzed, methods based on pain education, CBT or CFT, combined with various types of physiotherapeutic interventions, appear to be superior, with moderate evidence, to physiotherapeutic interventions in the medium term alone (range: 3 months to 1 year) in relation to pain relief and disability reduction in patients with CLBP.

In any case, it could be of a great help to new studies that focus on pain education, in conjunction with standardized physiotherapy treatment for the management of CLBP. Consequently, this latter treatment should ideally be reproduced on the control group, without, obviously, resorting to pain education techniques or cognitive-behavioral approaches. In this way, accurate conclusions can be drawn regarding the effects of implementing pain education in the management of patients with CLBP.

## Figures and Tables

**Table 1:** Search strings used.

Database	String Used	Note
Pubmed	(([pain education [MeSH Terms]) OR (cognitive behavior therapy [MeSH Terms])) AND (chronic low back pain [MeSH Terms])) AND (lumbar pain [MeSH Terms])	-
Scopus	(TITLE-ABS-KEY (*pain* AND *education*) OR TITLE-ABS-KEY (*cognitive* AND *behavior* AND *therapy*) AND TITLE-ABS-KEY (*chronic* AND *low* AND *back* AND *pain*))	-
Pedro	Abstract & title: pain education chronic low back pain; Method: clinical trial; Published since: 2011; Abstract & title: cognitive behavior therapy chronic low back pain; Method: clinical trial; Published since: 2011.	The results of the two researches were combined
Cochrane library	((Title abstract keyword: pain education) AND (Title abstract keyword: chronic low back pain)) with Publication Year from 2011 to 2021, with Cochrane Library publication date from Sep 2011 to Sep 2021, in Trials; ((Title abstract keyword cognitive behavior therapy) AND (Title abstract keyword: chronic low back pain)) with Publication Year from 2011 to 2021, with Cochrane Library publication date from Sep 2011 to Sep 2021, in Trials.	The results of the two researches were combined

**Table 2:** PEDro Scale. [11].

1. Eligibility criteria were specified	no ❑	yes ❑	where:
2. Subjects were randomly allocated to groups (in a crossover study, subjects were randomly allocated an order in which treatments were received)	no ❑	yes ❑	where:
3. Allocation was concealed	no ❑	yes ❑	where:
4. The groups were similar at baseline regarding the most important prognostic indicators	no ❑	yes ❑	where:
5. There was blinding of all subjects	no ❑	yes ❑	where:
6. There was blinding of all therapists who administered the therapy	no ❑	yes ❑	where:
7. There was blinding of all assessors who measured at least one key outcome	no ❑	yes ❑	where:
8. Measures of at least one key outcome were obtained from more than 85% of the subjects initially allocated to groups	no ❑	yes ❑	where:
9. All subjects for whom outcome measures were available received the treatment or control condition as allocated or, where this was not the case, data for at least one key outcome was analysed by “intention to treat”	no ❑	yes ❑	where:
10. The results of between-group statistical comparisons are reported for at least one key outcome	no ❑	yes ❑	where:
11. The study provides both point measures and measures of variability for at least one key outcome	no ❑	yes ❑	where:

**Table 3:** PEDro scale for each study.

	1-Eligibility Criteria	2-Randomization?	3-Hidden Assignment?	4-Homogeneity of the Groups?	5-Blindness of the Subjects?	6-Blindness of Therapists?	7-Blindness of the Evaluators?	8-Subject to Follow-Up?	9-Intention to Treat?	10-Statistical Comparison between Groups?	11-Measurements of Magnitude and Variability?	PEDro Score
GB Pardo et al. (2017) [12]	✓	✓	✓	✓	✗	✗	✗	✓	✗	✓	✓	**6/10**
DC Cherkin et al. (2016) [12] *	✓	✓	✓	✓	✗	✗	✗	✗	✗	✓	✓	**5/10**
JL Díaz-Cerrillo et al. (2015) [13] *	✓	✗	✗	✓	✗	✗	✓	✓	✗	✓	✓	**5/10**
B. Khodadad et al. (2019) [14]*	✓	✓	✗	✗	✗	✗	✗	✓	✗	✓	✓	**4/10**
A. Louw et al. (2016) [15]	✓	✓	✓	✓	✗	✗	✗	✓	✗	✓	✓	**6/10**
M. O’Keeffe et al., (2019) [16]	✓	✓	✓	✓	✗	✗	✗	✗	✓	✓	✓	**6/10**
MJ Petrozzi et al. (2019) [17]	✓	✓	✓	✓	✗	✗	✗	✓	✓	✓	✓	**7/10**
T. Pincus et al. (2015) [18]	✓	✓	✓	✗	✗	✗	✗	✗	✓	✗	✓	**4/10**
P. Rabiei, B. Sheikhi, A. Letafatkar (2021) [19]	✓	✓	✓	✓	✗	✗	✗	✓	✗	✓	✓	**6/10**
RMA Van Erp et al. (2019) [20]	✓	✓	✓	✓	✗	✗	✓	✗	✓	✓	✓	**7/10**
J. Semrau et al. (2021) [21]	✗	✓	✓	✓	✓	✗	✓	✗	✓	✓	✓	**8/10**
KV Fersum et al. (2013) [22]	✓	✓	✓	✓	✗	✗	✓	✗	✗	✓	✓	**5/10**
P. Wälti et al. (2015) [23]	✓	✓	✓	✓	✗	✗	✓	✓	✓	✓	✓	**8/10**

Legend: ✓ = criterion satisfied; ✗ = criterion not satisfied; * = articles for which the PEDro scale is not provided directly by the database, therefore, the questionnaire was filled in on the basis of what is reported in the articles.

**Table 4:** Level of evidence of the included studies.

Level of Evidence (LOE)	Study/I.	PEDro Score (Ps)
Level I	J. Semrau et al. (2021) [21] P. Wälti et al. (2015) [23]	Ps ≥ 8/10
Level II	GB Pardo et al. (2017) [11] DC Cherkin et al. (2016) [12] JL Díaz-Cerrillo et al. (2015) [13] A. Louw et al. (2016) [15] M. O’Keeffe et al., (2019) [16] MJ Petrozzi et al. (2019) [17] P. Rabiei, B. Sheikhi, A. Letafatkar (2021) [19] RMA Van Erp et al. (2019) [20] KV Fersum et al. (2013) [22]	5/10 ≤ Ps <8/10
Level III	B. Khodadad et al. (2019) [14] T. Pincus et al. (2015) [15]	Ps = 4/10
Level IV		-
Level V		-

**Table 5:** Summary of the articles included.

Author and Year of Publication	Study Design and Level of Evidence (LOE)	No. of Patients (n), Characteristics and Duration of Symptoms (DDS)	Groups, Intervention and Number of Treatments (NT)	Outcome	Evaluations and Follow-Up	Summary of the Results	PEDro SCORE
GB Pardo et al. (2017) [11]	TRC LOE: II	n = 56 Age (years): 20–75 DDS ≥ 6 months	Experimental group n = 28 Motor control exercises, stretching, aerobic exercises, PNE Control group n = 28 Motor control exercises, stretching, aerobic exercises NT: Two sessions of 30–50 min each month apart + home exercises during the follow-up	PAIN: NRS DISABILITY: RDQ	Baseline 1 month 3 months	PAIN: although an improvement was observed in both groups, a significant difference was noticed the experimental group at each follow-up point (NRS: −2.2; −2.93, −1.28; *p* < 0.001; d = 1.37) DISABILITY: the results obtained on the RDQ also show significant improvements in favor of the experimental group (RDQ: −2.7; −3.9, −1.4), *p* < 0.001; d = 1.15)	6/10
DC Cherkin et al. (2016) [12]	TRC LOE: II	n = 342 males = 117 females = 225 Age (years): 20–70 (average 49) DDS: 3 months–50 years (mean 7.3 years)	Experimental group n = 113 (CBT, pain education and its relationship with worries and emotional state, relapse prevention, maintenance of improvements, relaxation techniques, pain adaptation strategies) Control group n = 113 (Any physiotherapy treatment the participants wanted to carry out) NT: 2 h per week for 8 weeks	PAIN: BPB DISABILITY: mRDQ	Baseline 4 weeks 8 weeks 26 weeks 52 weeks	PAIN: in terms of BPB, the participants who improved most consistently were those of the experimental group (45%) versus those of the control group (27%). DISABILITY: significant improvements were observed at 26 weeks, on the mRDQ, in a percentage manner higher for the experimental group (58%) than for the control group (44%).	5/10
JL Díaz-Cerrillo et al. (2015) [13]	quasiTRC LOE: II	n = 128 Males = 51 Females = 77 Age (years): 18–65 DDS > 3 months	Experimental group n = 64 Functional education, cognitive-behavioral education: cognitive restructuring, goal reorientation and attention deviation Control group n = 64 Functional education, strengthening and stretching exercises of the spine, physical activity at home NT: 7	PAIN: NRS-11 DISABILITY: RDQ	Baseline Post intervention	Improvements were noted in both groups regarding the two outcome measures at the end of treatment. In addition, significant differences were observed between the two groups in favor of the experimental group as regards the reduction of the disability index, but not as regards the pain scale. PAIN: (*p* = 0.280) DISABILITY: (*p* = 0.046)	5/10
B. Khodadad et al. (2019) [14]	PRETEST-POSTTEST INTERVENTION LOE: III	n = 52 Age (years): mean 44.3 ± 2.46 VAS: 3/10–8/10 DDS > 3 months	Experimental group n = 17 CFT, pain physiology education, exercise, relaxation techniques, identification of incorrect movements and postures, aerobic exercise, stretching Control group n = 18 Traditional physiotherapy NT: Experimental group = Three sessions per week for 8 weeks Control group = not specified	PAIN: VAS	Baseline Post-surgery (8 weeks)	PAIN: An average decrease of 40% on the VAS was observed in the experimental group. No significant changes were observed for the same variable in the control group.	4/10
A. Louw et al. (2016) [15]	TRC LOE: II	n = 62 Females = 35 Males = 27 Age (years) > 18 (average 60.1) Mean age: 60.1 DDS> 6 months (mean 9.26 years)	Experimental group n = 33 Manual therapy techniques, Mulligan mobilizations Pain education, explanation of the mechanisms of neuroplasticity Control group n = 29 Manual therapy techniques, Mulligan mobilizations, explanation of the biomechanics of the lumbar spine NT: One session of 15 min (10 min of manual treatment + 5 min of explanation)	PAIN: NRS	Baseline Post intervention	PAIN: Neither group (experimental nor control) showed significant improvements on the NRS after the respective treatment sessions (Interaction effect *p* = 0.325) Experimental group: 3.8 ± 2.1 pre-treatment; 3.0 ± 2.4 post treatment. Control group: 4.3 ± 2.4 pre-treatment; 4.0 ± 2.5 post treatment.	6/10
M. O‘Keeffe et al., (2019) [16]	TRC LOE: II	n = 206 ODI score > 14% Age (years): 18–75 DDS ≥ 6 months	Experimental group n = 106 CFT, giving meaning to pain, pain control exposure, lifestyle change Control group n = 100 Exercise, education and relaxation NT: Experimental group = variable, on average five treatments in 6–8 weeks Control group = Six sessions in 6–8 weeks	PAIN: NRS DISABILITY: ODI	Baseline 6 months 12 months	PAIN: No obvious differences between groups were observed in pain intensity either at 6 months (mean difference: 0.76, −0.02 to 1.54; *p* = 0.056) or at 12 months (mean difference: 0.65, −0.20 to 1.50; *p* = 0.134). DISABILITY: the experimental group showed a more evident reduction in disability than the control group at 6 months (mean difference: 8.65, from 3.66 to 13.64; *p* = 0.001) and at 12 months (mean difference: 7.02, from 2.24 to 11.80; *p* = 0.004).	6/10
MJ Petrozzi et al. (2019) [17]	TRC LOE: II	n = 108 Age (years) > 18 average 50.4 ± 13.6 DDS > 3 months	Experimental group n = 54 Physiotherapy (manual therapy, exercise, education) CBT (information on negative emotions, cognitive-behavioral therapy; behavioral approach strategies) via MoodGYM software Control group n = 54 Physiotherapy (manual therapy, exercise, education) NT: Experimental group = mean 7.7 (SD 2.4) Control group = mean 7.7 (SD 2.0)	PAIN: NRS DISABILITY: RDQ	Baseline 8 weeks 6 months 12 months	PAIN: A moderate reduction in pain symptoms is observed for both groups at the end of treatment (8 weeks), although it is not effectively maintained during follow-up. DISABILITY: Significant improvements were observed in both groups at the end of treatment (8 weeks, and then maintained at 6 and 12 months), but without major differences between the two groups (*p* = 0.70) at each follow-up point.	7/10
T. Pincus et al. (2015) [18]	TRC LOE: III	n = 89 Males = 35 Females = 54 Age (years): mean 44.6 (SD 16.01) DDS > 3 months	Experimental group n = 45 CBT Control group n = 44 Physiotherapy NT: Eight sessions of 1 h	PAIN: BPI, CPAQ DISABILITY: RDQ	Baseline 3 months 6 months	PAIN: The average results on the pain acceptance scales were higher for the experimental group than for the control group (increase of 7.9 versus 5.1). DISABILITY: A change in the disability index at 6 months was greater in the experimental group than in the control group.	4/10
P. Rabiei, B. Sheikhi, A. Letafatkar (2021) [19]	TRC LOE: II	n = 73 DDS > 3 months	Experimental group n = 37 Neurophysiological education of pain, motor control exercises Control group n = 38 Conventional exercise NT: 16 (Two weekly sessions for 8 weeks)	PAIN: VAS DISABILITY: RDQ	Baseline 8 weeks	Both groups showed significant improvements under the two outcome measures examined, with the experimental group showing more significant improvements than the control group. ACHE: (*p* = 0.041, η*p*^2^ = 0.06) DISABILITY: (*p* = 0.021, η*p*^2^ = 0.07)	6/10
RMA Van Erp et al. (2019) [20]	TRC LOE: II	n = 25 Males = 11 Females = 14 Age (years): 18–62 (mean 44 (SD 12.2) DDS ≥ 12 weeks	Experimental group n = 12 Information on pain mechanisms, behavior and beliefs, coping strategies, goal-setting and self-management strategies, elements of CBT Control group n = 13 Usual treatment for low back pain NT: on average 8 (range 3–12)	PAIN: NRS DISABILITY: QBPD	Baseline Post intervention 3 months	PAIN: There were no significant differences between the two intervention groups at both endpoints. DISABILITY: No significant differences were found between the two groups after the intervention (mean difference 0.10, 95% CI: −12.9 to 13.1) and at follow-up (mean difference −5.4, 95% CI −19.1 to 8.3).	7/10
J. Semrau et al. (2021) [21]	TRC LOE: I.	n = 351 Age (years): Experimental group mean = 51.24 (SD 7.4) Control group mean = 51 (SD 7.4) DDS> 3 months	Experimental group n = 176 Behavioral exercises according to the BPS approach, coping strategies in relation to movement and low back pain episodes, education, maintenance of physical activity during the follow-up Control group n = 175 Standard exercises, physical activity NT: Experimental group = 15 sessions Control group = 13 sessions	PAIN: NRS DISABILITY: HFAQ	Baseline Post intervention 6 months 12 months	PAIN: There were no significant differences between the two groups on the NRS, either at the end of treatment or at the subsequent follow-up points, although modest improvements were observed in both groups. DISABILITY: There were no significant differences, neither at the end of the treatment sessions, nor at the subsequent follow-up points, with both study groups showing improvements on the HFAQ.	8/10
KV Fersum et al. (2013) [22]	TRC LOE: II	n = 121 Age (years): 18–65 ODI > 14%, NRS > 2/10 DDS > 3 months	Experimental group n = 62 CFT, functional education, physical activity Control group n = 59 Exercise, joint mobilization, manual therapy, applied to the spine or pelvis NT: Experimental group = 106 h of CB-CFT Control group = at the discretion of the physiotherapist	PAIN: NRS DISABILITY: ODI	Baseline Post-surgery (3 months) 12 months	The experimental group showed more significant improvements in both pain and disability, post-surgery and following the insane up. PAIN: The experimental group improved on average by 3.2 points on the NRS, and the control group by 1.5 points. DISABILITY: At 12 months, the experimental group showed an average improvement of 13.7 percentage points on the ODI scale, while the control group showed an improvement of 5.5%.	5/10
P. Wälti et al. (2015) [23]	TRC LOE: I.	n = 28 Males = 13 Females = 15 Age (years): 18–60 average 41.5 (Ds 10.6) DDS ≥ 3 months	Experimental group n = 14 Pain neurophysiology education, motor sense training for the trunk, trunk control exercises, home training during follow-up Control group n = 14 Conventional physiotherapy, functional education, home training during follow-up NT: One or two sessions per week, for 8 weeks (maximum 16 sessions)	PAIN: NRS DISABILITY: RDQ	Baseline 12 weeks	PAIN: A reduction in pain intensity was recorded both in the experimental group 2.14 (1.0 to 3.5) and in the control group (0.69, −2.0 to 2.5), with a moderate difference in favor of the experimental group DISABILITY: The reduction in the disability index, found in both groups, does not reveal significant differences in favor of one or the other. (Experimental group: 6.71, 4.2–9.3 Control group: 4.69, 1.9–7.4)	8/10

Abbreviations: TRC = Controlled Randomized Trial, VAS = Visual-Analog Scale (0–10) (PAIN), CBT = Cognitive Behavioral Therapy, RDQ = Roland Disability Questionnaire (0–24) (DISABILITY), mRDQ = modified Roland Disability Questionnaire (0–23) (DISABILITY), BPB = Back pain bothersomeness (0–10) (PAIN), ODI = Oswestry Disability Index (DISABILITY), NRS = Numeric Rating Scale (0–10) (PAIN), CFT = Cognitive Functional Therapy, *p* = *p*-value, PNE = Pain Neurophysiology Education, BPI = Brief Pain Inventory, CPAQ = Chronic Pain Acceptance Questionnaire, BPS = bio-psycho-social, HFAQ = Hannover Functional Ability Questionnaire (1–100), QBPD = Quebec Back Pain Disability Score. NT = Number of treatments. LOE: Level of Evidence.

## Data Availability

Not applicable.
